# The effect of spread through air spaces on postoperative recurrence-free survival in patients with multiple primary lung cancers

**DOI:** 10.1186/s12957-024-03351-3

**Published:** 2024-03-05

**Authors:** Hongsheng Xie, Shihua Dou, Xiaoxiang Huang, Yuxin Wen, Lin Yang

**Affiliations:** 1 The Second Clinical Medical College, Jinan University, Shenzhen, Guangdong China; 2 Department of Thoracic Surgery, the First Affiliated Hospital of Hainan Medical College, Haikou, China; 3 Department of Thoracic surgery, Shenzhen People’s Hospital (The Second Clinical Medical College, Jinan University, The First Affiliated Hospital, Southern University of Science and Technology), Shenzhen, Guangdong China

**Keywords:** Spread through air spaces, Multiple primary lung cancer, Recurrence-free survival, Retrospective cohort study

## Abstract

**Purpose:**

The purpose of the study was to investigate the effect of spread through air spaces (STAS) on the postoperative prognosis of patients with multiple primary lung cancers staged from IA to IB based on tumor size.

**Methods:**

Clinicopathological and follow-up data of 122 patients with multiple primary lung cancers diagnosed at stages IA-IB and surgically treated at the Department of Thoracic Surgery, Shenzhen people’s Hospital from January 2019 to December 2021 were retrospectively analyzed. The study involved 42 males and 80 females. STAS status was used to divide them into two groups (87 cases in STAS (-) and 35 cases in STAS (+)). A logistic regression analysis, univariate and multivariate Cox regression analysis, and Kaplan-Meier curves (K-M) were used to determine how STAS affected recurrence-free survival (RFS) in patients.

**Results:**

STAS (+) had a significantly higher recurrence rate than STAS (-). STAS was predicted by smoking history (*P* = 0.044), main tumor diameter (*P* = 0.02), and solid nodules on chest CT (*P* = 0.02). STAS incidence was not significantly different between lobectomy and sublobar resection groups (*P* = 0.17). Solid nodules on CT, tumor diameter, vascular invasion, pleural invasion, and STAS were significant predictors of recurrence in the univariate Cox regression analysis. Tumor diameter, pleural invasion and STAS were significant prognostic factors for recurrence in the multivariate Cox regression analysis. Furthermore, STAS (+) group was at greater risk of recurrence than STAS (-) group (34% vs. 0%, *P* < 0.05)。.

**Conclusion:**

Stage IA-IB multiple primary lung cancer patients with STAS (+) had a higher recurrence rate and a shorter overall survival rate.

## Introduction

As reported in the Global Cancer Report of 2022, the mortality rates for cancer have been consistently declining, with lung cancer showing the most significant decrease in mortality rates (In the past 30 years, lung cancer mortality rates have decreased by 56% in males and 32% in females). Even though lung cancer ranks among the most common cancer-related deaths globally, early detection and treatment are still vital [1]. More and more patients with lung nodules and even early-stage lung cancer are being detected by low-dose high-resolution computed tomography (CT) scans, increasing public awareness of multiple primary lung cancer (MPLC). The MPLC is the occurrence of two or more primary lung cancers in the same individual, either simultaneously or sequentially, located either in the same lung or in the contralateral lung, and they may have the same or different histological types [2]. MPLC is considered an early-stage lung cancer, and its prognosis is generally better, with surgical resection being the primary treatment modality [3].

The invasive behavior of lung cancer includes: (1) non-lepidic histological growth pattern; (2) fibroblastic proliferation with connective tissue growth; (3) invasion of blood vessels or the pleura. However, in 2015, the World Health Organization identified a fourth invasive behavior of lung cancer, spread through air space (STAS). The term is described as “A micropapillary cluster, compact group, or a single cancer cell spreads within air spaces beyond the initial tumor boundary into surrounding lung tissue.” [4]. The incidence of STAS in diagnosed lung cancer ranges from 15 to 73% based on relevant literature [5–8]. In meta-analyses, STAS has been demonstrated to be an important adverse prognostic factor for patients receiving surgical removal of lung cancer [9–11]. Furthermore, multiple studies have indicated a strong correlation between STAS and patient survival and prognosis, with STAS-positive patients exhibiting significantly shorter recurrence-free survival (RFS) compared to STAS-negative patients [12–14]. However, STAS is effective in the early days, whether the effect of RFS in lung adenocarcinoma is due to its concomitant adverse prognostic features and the different pathological diagnoses of tumor size for early lung adenocarcinoma has an effect.

To determine how STAS plays a role in MPLC prognosis and recurrence, our study examined the influence of STAS on the recurrence and metastasis of patients with multiple primary lung cancers based on a previously collected database.

## Materials and methods

### Database

It is a retrospective study based on the clinical medical database of Shenzhen People’s Hospital. Clinical and pathological TNM staging (8th edition of the International Union Against Cancer staging system) was used for staging. Data were collected on the following parameters: (1) patient characteristics like age, gender, smoking history, etc.; (2) diagnostic information, including diagnostic methods, thin-layer chest CT(The slice thickness of the CT was 1 mm), etc.; (3) surgical procedures, including lobectomy, segmentectomy, or wedge resection, etc.; (4) pathological findings, including size, grading, lymph node metastases, STAS, lymphatic vessel invasion, and pleural invasion, etc.; (5) results, including recurrence, mortality, and stability.

### Data collection

General information: collected through the electronic medical record system, including surgical age, gender, smoking history, history of malignant tumors, and surgical approach.

Imaging information: collected from preoperative staging examinations, including chest CT, abdominal and urological ultrasound, positron emission tomography/computed tomography, bone scan.

Postoperative pathological information: Two pathologists with more than 3 years’ experience reviewed the slides. This includes tumor size, number of lesions, histological grade, adenocarcinoma subtypes, STAS (spread through air spaces), presence of mucinous adenocarcinoma, pleural invasion, vascular invasion, and neural invasion. In accordance with the 8th edition of the TNM staging system, we performed histological subtyping, grading, and pathological staging.

### Patients

In the period January 2019 to December 2021, 122 patients diagnosed with lung cancer underwent lung cancer surgery .Inclusion criteria were as follows: (1) A single-center clinical and pathological study of patients who are eligible for surgery between 2019 and 2021 based on AJCC TNM staging (8th edition) for stage IA-IB non-small cell lung cancer (NSCLC ); (2) confirmation of NSCLC in at least two or more lesions by postoperative histology or cytology; (3) availability of complete preoperative imaging data and postoperative pathological reports; (4) availability of comprehensive follow-up information for the patients. The exclusion criteria were as follows: (1) evidence of intrapulmonary or distant lymph node metastasis based on postoperative pathology; (2) presence of concurrent malignancies in the patients; (3) coexistence of other serious diseases that may affect follow-up and significantly impact short-term survival; (4) receiving antitumor treatments such as chemotherapy, targeted therapy, or immunotherapy before surgery. MPLC was diagnosed based on the following criteria: (1) One or more lesions are adenocarcinomas in situ or minimally invasive adenocarcinomas; (2) presence of different histological types between the lesions; (3) presence of the same histological type with distinct cytological and stromal features among the lesions; (4) presence of the same histological type with consistent primary components (such as papillary or acinar) but differing in cytological and stromal features [15].

### Statistical analysis

Various categorical and continuous variables were evaluated using Student’s t-tests. STAS was predicted by logistic regression analysis. All patients were followed up until September 2023. Time between surgery date and last appointment or recurrence was the primary endpoint. Recurrence-free survival was estimated using Kaplan-Meier methods. Prognostic factors were determined using a multivariate Cox proportional hazards regression analysis. Data analysis was performed using SPSS 26.0 software to assess survival differences. *P*-values less than 0.05 were considered statistically significant.

## Results

A median age of 58 was observed at surgery (range 28 to 80). In total, 122 patients were enrolled, 42 of whom were males, and 80 of whom were females. There were 102 non-smokers (83.6%) and 20 smokers (16.4%). There were 113 cases in stage IA (92.7%) and 9 cases in stage IB (7.3%). There were 12 cases of vascular invasion (9.8%) and 13 cases of pleural invasion (10.6%). STAS was found in 35 cases (28.7%). Table 1 provides a summary of patients with STAS. In STAS patients, smokers, stage IB, solid nodules, vascular invasion, and pleural invasion was significantly higher. Multivariate analysis of factors influencing STAS before surgery (Table 2) showed that smoking history (*P* = 0.044), size of the main tumor diameter (*P* = 0.02), and solid nodules on chest CT (*P* = 0.02) were predictive factors for STAS.

**Table 1:** Patient characteristics

Factors	STAS (+) *n* = 35	STAS (−) *n* = 87	*P*
Male/female	15/20	27/60	0.001
Median age (years)	62	55	0.003
Non-smoker/smoker	25/10	77/10	0.02
Lobectomy/ Sublobectomy	22/13	41/46	0.1
p-stage IA/IB	28/7	85/2	0.003
Median tumor size (cm)	2.1	1.3	0.003
Solid nodules on CT	29	31	<0.001
LV invasion (+/−)	11/24	1/86	<0.001
Pleural invasion (+/−)	12/23	2/85	<0.001

STAS: spread through air spaces

Sublobectomy: segmentectomy or wedge resection

Median tumor size (primary lesion)

LV: lymph vascular

The χ2 test was used for categorical data. Student’s t-test or the Wilcoxon rank-sum test was used for continuous data

**Table 2:** Predictive factors for STAS

Preoperative variables	OR	95% CI	*P*
Male	1.89	0.50–7.18	0.35
Age	1.02	0.97–1.07	0.43
Smoker	0.19	0.04–0.96	0.044
Tumor size	3.44	1.57–7.56	0.002
Solid nodules on CT	0.18	0.06–0.52	0.002

STAS: spread through air spaces

Tumor size (primary lesion)

CI: confidence interval

Logistic regression analysis

**Fig. 1:**
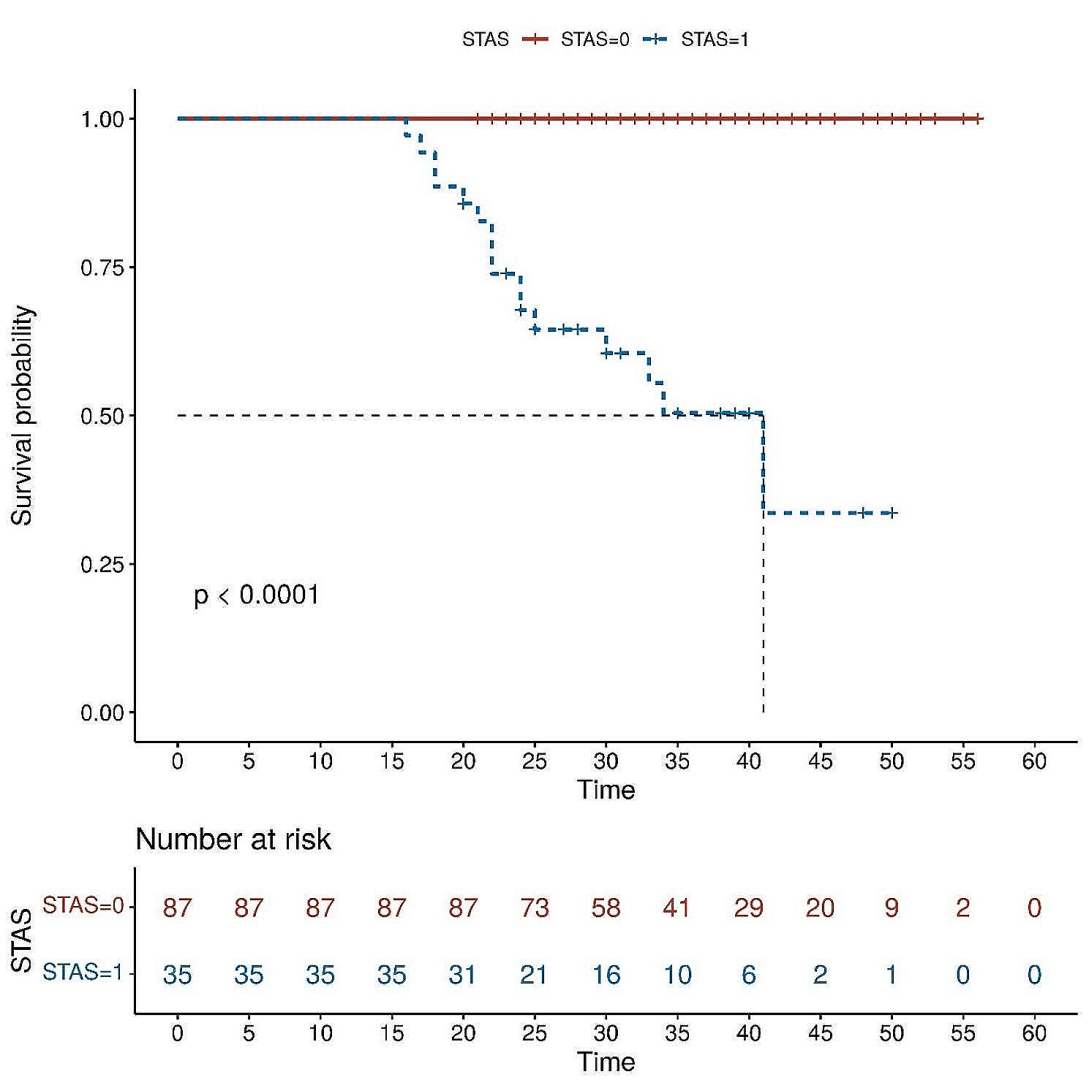
Recurrence-free survival according to the presence of spread through air spaces. Survival differences were assessed by the log-rank test

**Fig. 2:**
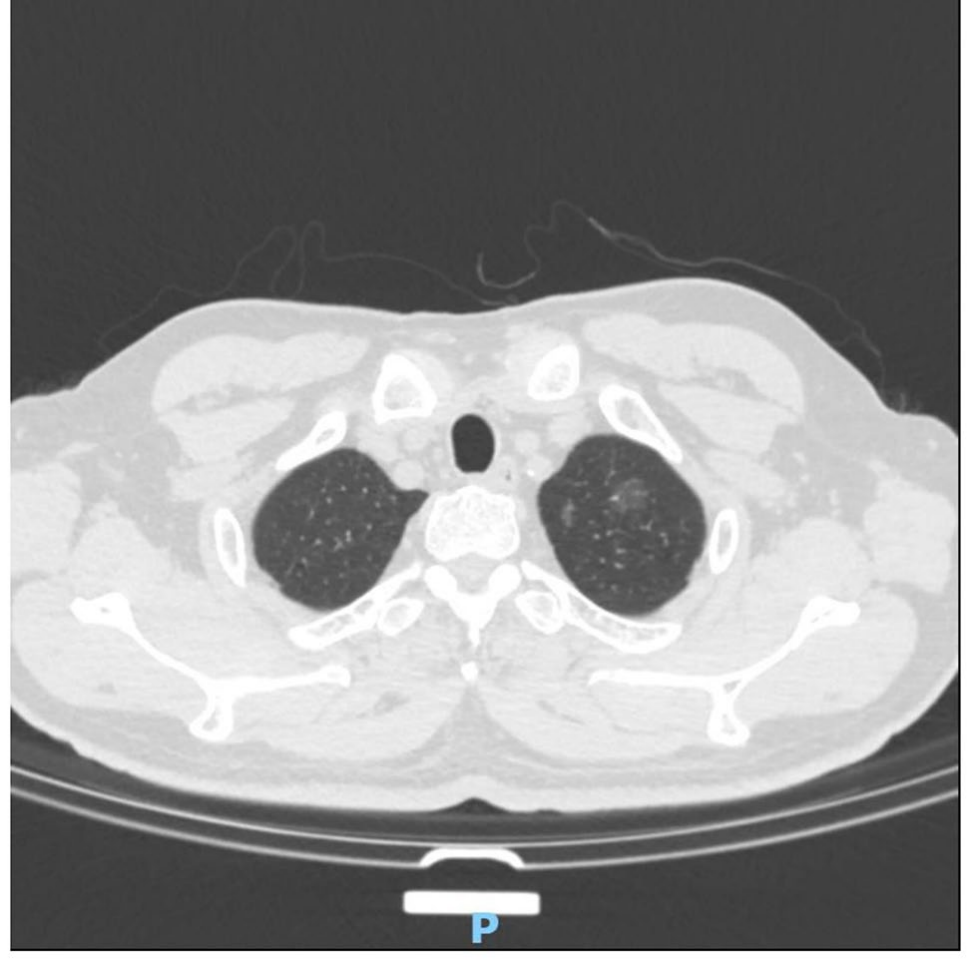
This is a case of multiple pulmonary nodules with pGGO

The 122 patients received 63 (51.6%) lobectomies and 59 (48.4%) sublobar resections (segmentectomy or wedge resection). Neither lobectomy nor sublobar resection showed a statistically significant difference in STAS incidence (*P* = 0.17). After lung cancer surgery, among the 122 patients, there were 12 cases of recurrence (10%) and 1 death (0.8%), all of which were in the STAS (+) group. The recurrence rate was 4.8% for lobectomy and 5.1% for sublobar resection, and there was no statistically significant difference between surgical procedures in terms of recurrence rates (*P* = 0.9). The sites of recurrence included 12 cases in the lungs, 2 cases in the bones, 3 cases in the lymph nodes, 1 case in the kidney, and 1 case in the pancreas. Based on Kaplan-Meier estimates, median follow-up was 33 months (95% confidence interval, 20–56 months). Overall recurrence-free survival was 65.7%. In univariate analysis, solid nodules on CT, tumor diameter, vascular invasion, pleural invasion, and STAS were significant predictors of recurrence (Table 3). Kaplan-Meier curve for recurrence-free survival is shown in Fig. 1; Recurrence-free survival was 65.7% for STAS positive patients and 100% for STAS negative patients (*P*<0 0.001). In the multivariate Cox regression analysis, tumor diameter, pleural invasion, and STAS played significant roles in predicting recurrence. (Table 3). Figure 2, 3 illustrates the CT images of three enrolled patients, and Figs. 4, 5 and 6 illustrates the pathological images of STAS of one enrolled patient.

**Table 3:** Prognostic factors for recurrence-free survival

variables	Univariate analysis			Multivariate analysis		
	95%CI	HR	*P*	95%CI	HR	*P*
Male Age Smoker Lobectomy Solid nodules on CT Tumor size	0.25–1.78 0.95–1.11 0.15–1.26 0.50–15.10 0.001–0.67 1.18–1.69	0.66 1.03 0.44 2.74 0.01 1.41	0.41 0.44 0.13 0.25 0.03 0.001	0.20–2.63 0.92–1.09 0.49–5.45 0.84–18.73 0.001–0.82 0.74–1.62	0.72 1.00 1.63 3.96 0.15 1.10	0.62 0.54 0.43 0.08 0.08 0.02
LV invasion(+)	0.03–0.23	0.08	0.001	0.60–7.16	2.06	0.25
Pleural Invasion(+)	0.02–0.15	0.05	0.001	1.58–13.16	4.56	0.001
STAS(+)	0.001–0.98	0.002	0.04	0.001–1.52	0.5	0.03

CI: confidence interval

Tumor size (primary lesion)

LV: lymph vascular

STAS: spread through air spaces

Cox proportional hazards model

**Fig. 3:**
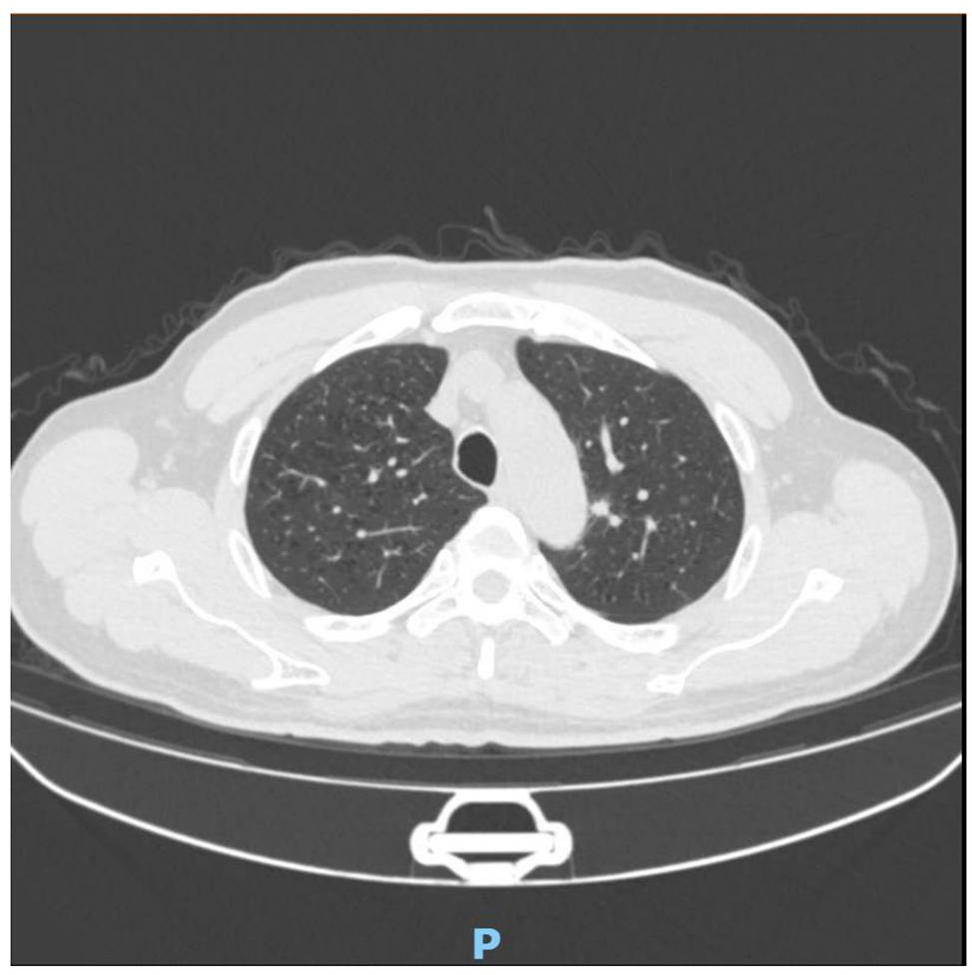
This is a case of multiple pulmonary nodules with PSN

**Fig. 4:**
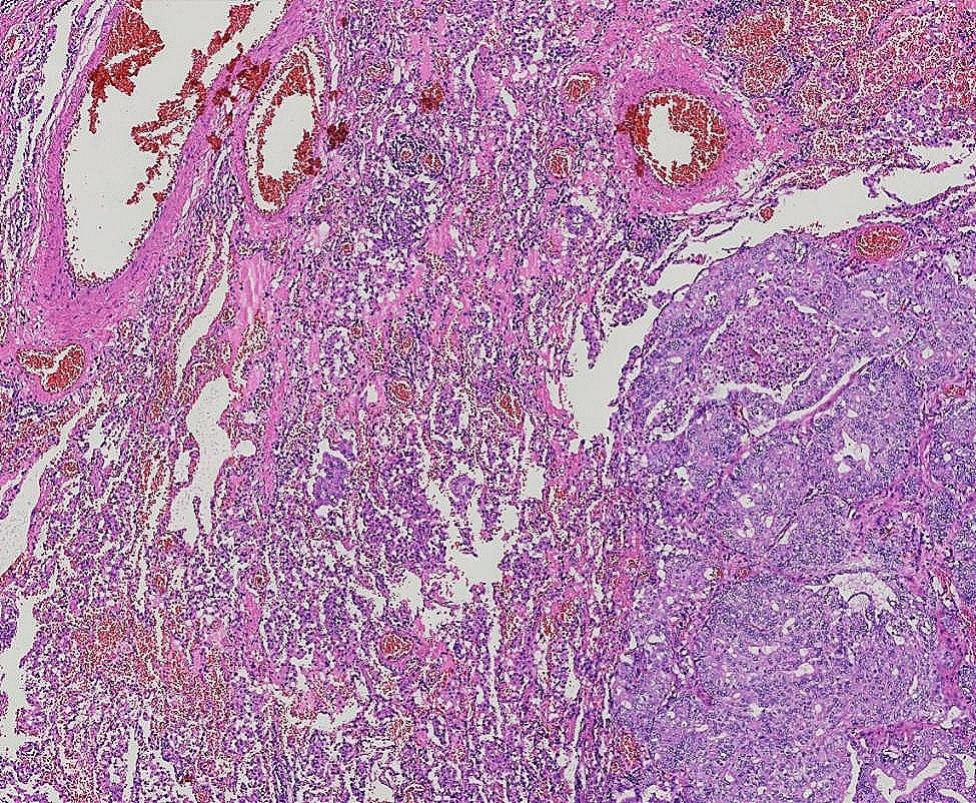
STAS×40

**Fig. 5:**
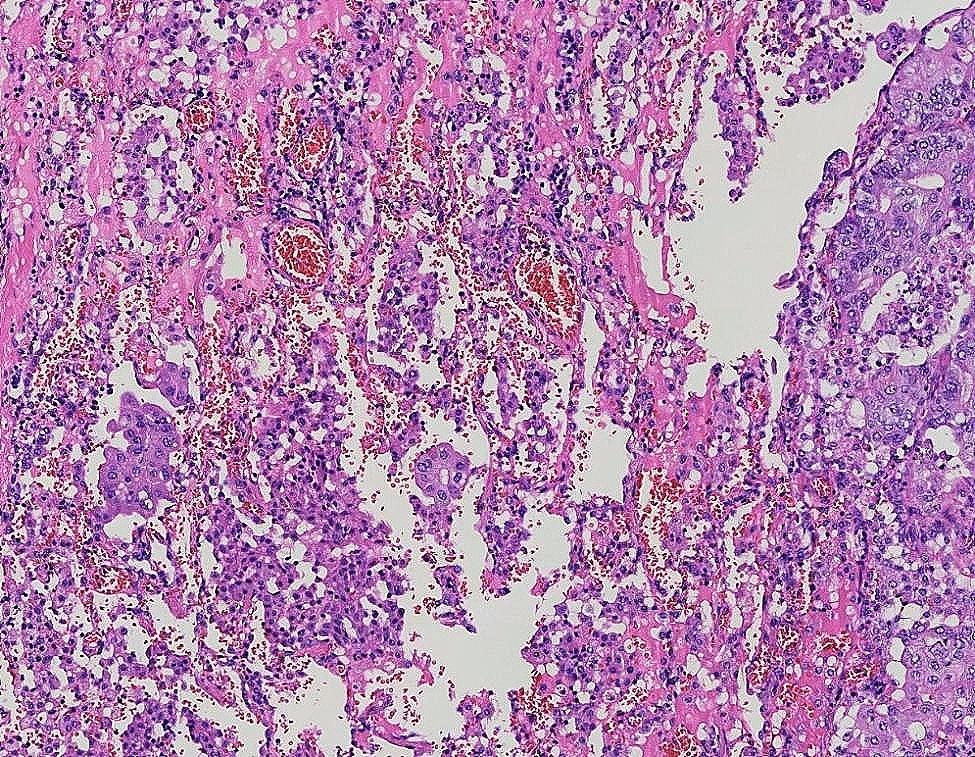
STAS×100

**Fig. 6:**
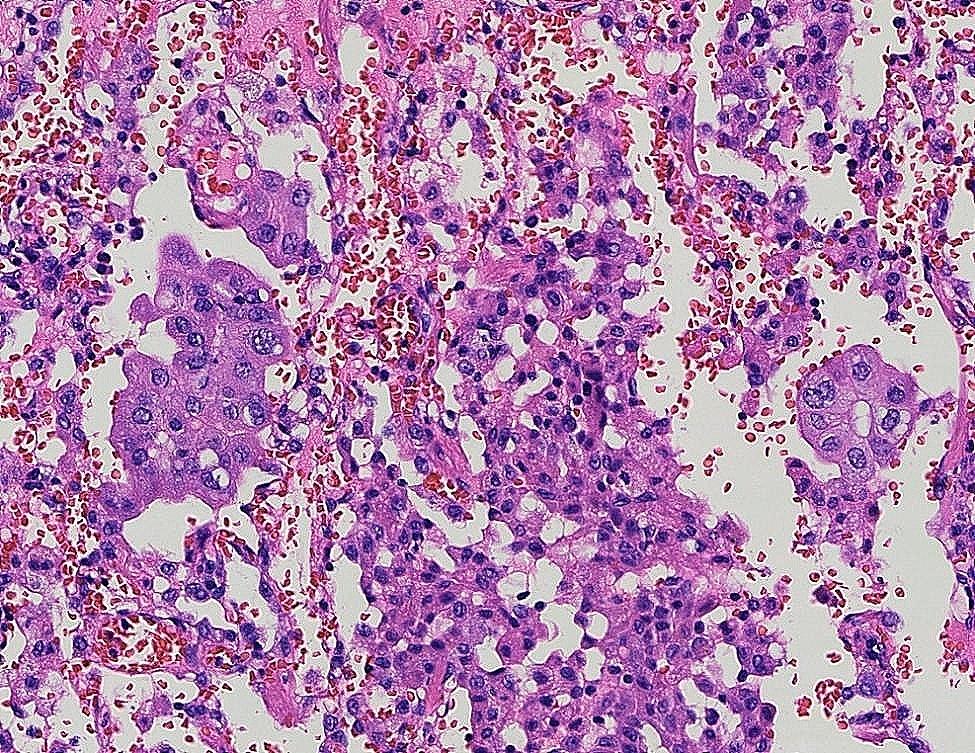
STAS×200

## Discussion

The study indicates that in MPLC patients, the STAS (+) group is more prone to recurrence compared to the STAS (-) group. Smoking history, size of the main tumor, and solid nodules on chest CT are predictive factors for STAS. Sublobar resection and lung lobectomy did not differ statistically in STAS incidence.

Multiple primary lung cancers are commonly adenocarcinomas, and distinguishing them from intrapulmonary metastases (IM) is often a concern for clinicians. In comparison to intrapulmonary metastases, multiple primary lung cancers generally have a better prognosis and a greater response to surgical resection, while intrapulmonary metastases are often advanced lesions with limited surgical options and are primarily treated with radiation, chemotherapy, and targeted therapy.

In terms of surgical approach, it is generally accepted that the physical squeezing pressure during surgery can affect the presence of STAS, especially in thoracoscopic surgery, when the specimen is removed through a tiny thoracoscopic hole. However, Blaauwgeers et al. were preceded by a prospective study [16]. It was shown that there was no difference in the incidence of loose fragments between different surgical groups (thoracotomy versus thoracoscopic surgery). Since STAS is associated with a higher ADC stage, the outcome may be due to the possibility that patients with a higher ADC stage may undergo radical surgery and thoracotomy. Whether STAS is a real in vivo phenomenon or an in vitro artifact is debatable. In thoracoscopic surgery, the excised lung specimen containing the tumor is squeezed through a tiny thoracoscopic foramen, which may cause tumor cells to detach around the tumor and move to adjacent air spaces [17]. In the current study, STAS is more common when thoracotomy is performed. In addition, numerous studies have shown that [18–20], STAS is independently associated with poor prognosis in patients with ADC. These arguments support that STAS is a real, important biological phenomenon and not a false artificial artifact.

Despite undergoing surgical treatment, 6.3-63.7% of MPLC patients still experience recurrence, suggesting the need to consider other risk factors for recurrence in addition to clinical staging [21]. Dai et al. suggested that STAS has a minimal impact on the prognosis of lung adenocarcinoma with a diameter < 2 cm, while STAS can significantly worsen the prognosis of patients with lung adenocarcinomas with diameters of 2–3 cm [22]. However, Kadota et al. found that STAS contributed independently to recurrence in lung adenocarcinoma with a 2 cm tumor diameter [23]. The studies primarily focused on early-stage lung adenocarcinoma, with limited experimental data regarding the relationship between STAS and MPLC.

Some studies propose that STAS is associated with epithelial-mesenchymal transition [24], which involves the loss of E-cadherin leading to decreased cell adhesion, promoting invasion and playing a role in tumor metastasis through lymphatic vessels, blood vessels, pleura, and STAS [25]. This mechanism may increase the likelihood of occult extrapulmonary metastasis during lung cancer surgery, increasing the risk of local and distant recurrence. STAS and its relationship with poor prognosis, particularly its association with IM, require further comprehensive research.

Moreover, considering that patients with multiple lung cancers require a greater extent of lung tissue resection, sublobar resection could be considered as the primary surgical approach to better preserve lung function. Suzuki et al. defined non-invasive lung cancer on imaging (tumor ≤ 2 cm, CTR ≤ 0.25) and found that sublobar resection with adequate margins (defined as at least 5 mm in the study) achieved close to 100% 5-year recurrence-free survival, with lower complication rates and less impact on lung function [26]. The studies primarily focused on patients with solitary lung cancer, and future research can focus on surgical approaches for multiple lung cancers.

## Limitations

This study is based on comprehensive clinical follow-up data and provides a certain reference basis for clinical determination of diagnosis and treatment plans. However, it still has certain limitations. In this study, patients were followed up for only 3 years; Further surveillance of survival rates over five years and recurrence-free survival rates is needed. Moreover, this is a single-center retrospective study and the number of cases is little, which may have biases in patient selection. Further multicenter studies with larger sample sizes should be considered in the future.

## Conclusions

In summary, in patients with MPLC, STAS is predominantly observed in invasive cases and is strongly associated with adverse prognosis and high rates of recurrence.

## Data Availability

No datasets were generated or analysed during the current study.
